# Event and Comment

**Published:** 1911-04-15

**Authors:** 


### DENTAL REGISTER.

**Vol. LXV. April 15, 1911. No. 4**

### EVENT AND COMMENT.

"MUSH BITE." We encounter frequently in reading current literature this expression. It seems so meaningless and inappropriate that we want to register a protest against its use. The term is always applied to the method used by hurried dentists in securing the articular relations of the jaws, by having the patient bite into a piece of soft wax placed between the upper and lower teeth or edentulous jaws. The method is faulty enough without handicapping it still further with a meaningless name. If any term must be used to designate the process it should with propriety be called a "mash bite," which would give one an idea of the process and at the same time be a dignified and appropriate name. There can be no propriety in comparing bees wax with mush, as these materials are very different in their physical properties, and patients do not mush their teeth into the wax, but they do mash them into it.

"GET OUT OF THE SHOP." In a communication to the March *Western Dental Journal*, Dr. William H. Trueman deplores existing conditions which he sees. In spite of the fact that a higher standard of education is now required for admission to our dental colleges, that contributions to our current literature do not show a corresponding increase in breadth of scholarship. He admits that we have increased in technical skill, but are woefully lacking in general literary and scientific culture, and intimates that as a profession we can not even converse with men in other professions without shame to ourselves because of a lack of knowledge of general cultural topics.

Now all of this is a serious arraignment of our professional standards and educational methods, if not also of our professional spirit. Dr. Trueman has seen the greater part of our professional development and kept in close contact with it, particularly as it has found expression in its literature, and his matured conclusions are entitled to much consideration; but we question somewhat both his premise and conclusions, not because we know, but because we trust they may not embody a careful and comprehensive consideration of so important a matter. It is too large a subject for us to make, in this place, more than a suggestion that here is a field of research that some one who is capable and has the time and inclination should cultivate and develop: the exact facts that will convince tho e in control of our educational institutions and other organizations which are supposed to be doing all they can for the elevation of the profession, that they will have to either change their methods or make decided improvements in their administration.

As we view the profession generally, we believe with Dr. Trueman that technical development has almost run riot with us; but, at the same time there has of necessity followed a literary progress that should not be wholly ignored. There never was so much writing as to-day. It is true, much of it is not classical in attainment, or even in type. Have we had time enough to develop Dr. Trueman's ideals? Have our colleges even to-day entrance requirements that encourage us to expect better things in the near future?

"ZAHNAERZTE HAUS." In our announcement Department, we print the call for contributions to a professional library, research laboratory and meeting place for dentists in Berlin, Germany. This is a move in the right direction and should have the cordial support of any one having material suitable for the purpose. How much longer shall American dentists wait before taking up some such plan?

